# Fore-aft resistance applied at the center of mass using a novel robotic interface proportionately increases propulsive force generation in healthy nonimpaired individuals walking at a constant speed

**DOI:** 10.1186/s12984-019-0577-x

**Published:** 2019-09-06

**Authors:** Avantika Naidu, Sarah A. Graham, David A. Brown

**Affiliations:** 1 Program in Rehabilitation Sciences, Departments of Physical & Occupational Therapy, School of Health Professions, University of Alabama at Birmingham, 1716 9th Avenue South, Birmingham, AL 35233 USA; 2 Department of Physical Medicine and Rehabilitation, Harvard Medical School, Spaulding Rehabilitation Hospital, 300 First Avenue, Boston, MA, 02129, 1575 Cambridge St, Cambridge, MA 02138 USA; 3 University of California San Diego, School of Health Sciences, 9500 Gilman Drive, La Jolla, CA 92093-0012 USA; 4 The University of Texas Medical Branch, School of Health Professions, 301 University Blvd, Galveston, TX 77555-0128 USA

**Keywords:** Walking, Fore-aft resistance, Propulsion, Biomechanics, Treadmill-interface nonimpaired

## Abstract

**Background:**

Past studies have utilized external interfaces like resistive bands and motor-generated pulling systems to increase limb propulsion during walking on a motorized treadmill. However, assessing changes in limb propulsion against increasing resistance demands during self-controlled walking has not been undertaken.

**Purpose:**

We assessed limb propulsion against increasing fore-aft loading demands by applying graded fore-aft (FA) resistance at the center of mass during walking in a novel, intent-driven treadmill environment that allowed participants to control their walking speeds. We hypothesized that to maintain a target speed against progressively increasing resistance, participants would proportionately increase their limb propulsion without increasing vertical force production, with accompanying increases in trailing limb angle and positive joint work.

**Methods:**

Seventeen healthy-nonimpaired participants (mean age 52 yrs., SD = 11) walked at a target, self-controlled speed of 1.0 m/s against 10, 15, 20, and 25% (% body weight) FA resistance levels. We primarily assessed linear slope values across FA resistance levels for mean propulsive force and impulse and vertical impulse of the dominant limb using one-sample *t*-tests. We further assessed changes in trailing and leading limb angles and joint work using one-way ANOVAs.

**Results:**

Participants maintained their target velocity within an a priori defined acceptable range of 1.0 m/s ± 0.2. They significantly increased propulsion proportional to FA resistance (propulsive force mean slope = 2.45, SD = 0.7, *t* (16) =14.44, *p* < 0.01; and propulsive impulse mean slope = 0.7, SD = 0.25, *t* (16) = 11.84, *p* < 0.01), but had no changes in vertical impulse (mean slope = − 0.04, SD =0.17, *p* > 0.05) across FA resistance levels. Mean trailing limb angle increased from 24.3° at 10% resistance to 27.4° at 25% (*p* < 0.05); leading limb angle decreased from − 18.4° to − 12.6° (*p* < 0.05). We also observed increases in total positive limb work (F (1.7, 26) = 16.88, *p* ≤ 0.001, η^2^ = 0.5), primarily attributed to the hip and ankle joints.

**Conclusions:**

FA resistance applied during self-driven walking resulted in increased propulsive-force output of healthy-nonimpaired individuals with accompanying biomechanical changes that facilitated greater limb propulsion. Future rehabilitation interventions for neurological populations may be able to utilize this principle to design task-specific interventions like progressive strength training and workload manipulation during aerobic training for improving walking function.

**Electronic supplementary material:**

The online version of this article (10.1186/s12984-019-0577-x) contains supplementary material, which is available to authorized users.

## Introduction

Walking is a complicated motor task that requires generation of lower-limb muscle forces that both propel and vertically support the body, on a step-by-step basis [1]. During walking, proprioceptive limb-loading feedback via the Golgi-tendon organs (GTOs) and cutaneous sole receptors helps in regulating vertical support mechanisms (e.g., limb extension) from initial to mid stance, and propulsive force generation function from mid to terminal stance [2–4]. From a mechanical perspective, propulsive forces are the summation of the positive fore-aft component of the ground reaction force (GRF) vector, from mid to late stance, required to move the body’s center of mass (COM) forward in the sagittal plane [5]. Similar generation of propulsive forces by each limb helps maintain interlimb symmetry, walking speed, and efficiency [6].

Considering the important role of stance-phase proprioceptive feedback on locomotor regulation, it is not surprising that various studies have investigated the effects of altering limb-loading dynamics on propulsive-force generation, walking speed, and walking energetics [4, 7–14]. Among these, some have focused on altering vertical-loading demands at constant and varying walking speeds using body-weight support (BWS) [8–10, 12–14], or reduced gravity environments [15, 16], to highlight how reducing body-weight demands decreases propulsive-force generation during walking. Other studies have used both invasive and noninvasive procedures to demonstrate how reducing limb-loading proprioceptive feedback during walking decreases plantarflexor activity and propulsion generation during the second half of stance [4, 17]. Given that propulsive forces are generated in the fore-aft direction (anterior to posterior), remarkably few studies have explored the effects of altering stance phase fore-aft limb-loading demands without altering vertical-loading demands during walking [9]. These studies have mainly examined the effects of backward-directed resistance applied at the COM [9, 11, 18–21] while walking at constant speeds, or on an uphill incline [9, 22–26], on increasing propulsion. However, participants in these studies walked on machine-driven treadmills programmed at constant speeds, which decrease muscle-force generation requirements to maintain speed, evidenced by attenuated braking and propulsive force profiles. [27] Thus, investigation of walking function in such automated environments is not optimal to gauge the effects of altered loading feedback on propulsive-force generation required to maintain speed.

Taking advantage of a unique robotic treadmill interface that allows individuals to control their walking speeds, we explored the effects of increasing fore-aft loading demands by using the interface to apply graded fore-aft (FA) resistance at the COM in specific percentages of vertical body weight. We hypothesized that to maintain a target walking speed against progressively increasing levels of FA resistance: 1) healthy-nonimpaired participants would proportionately increase propulsion with increasing FA resistance, based on the expectation of symmetrical force production in healthy-nonimpaired individuals and previous literature demonstrating increases in propulsion in similar environments [9–11, 18], without altering their vertical-force production, due to the selective provision of forces in the fore-aft direction (i.e., no alteration of body weight); and 2) increases in propulsion would correspond with increases in trailing limb angle and total positive limb work given that both of these variables have been linked to propulsive force output [5, 28, 29].

## Methods

### Participants

Seventeen healthy, nonimpaired individuals (9 females), mean weight 179 lbs. (SD = 37), mean age 52 years (SD = 11) participated in this study after providing informed consent, approved by the Institutional Review Board of the University of Alabama at Birmingham. We assessed safety for participation in low-to-moderate exercise using the Physical Activity Readiness Questionnaire (PAR-Q) form. We excluded participants with a history of severe cardiovascular, neurological, or musculoskeletal disorders that could affect their walking function or ability to perform mild physical activity. We assessed limb dominance by asking participants which limb they would use to stand on one leg, and baseline heart rate, blood pressure, and self-selected comfortable walking speed (10-m walk test, mean speed = 1.2 m/s (SD = 0.03)) prior to study participation.

### Experimental environment

**Fig. 1:**
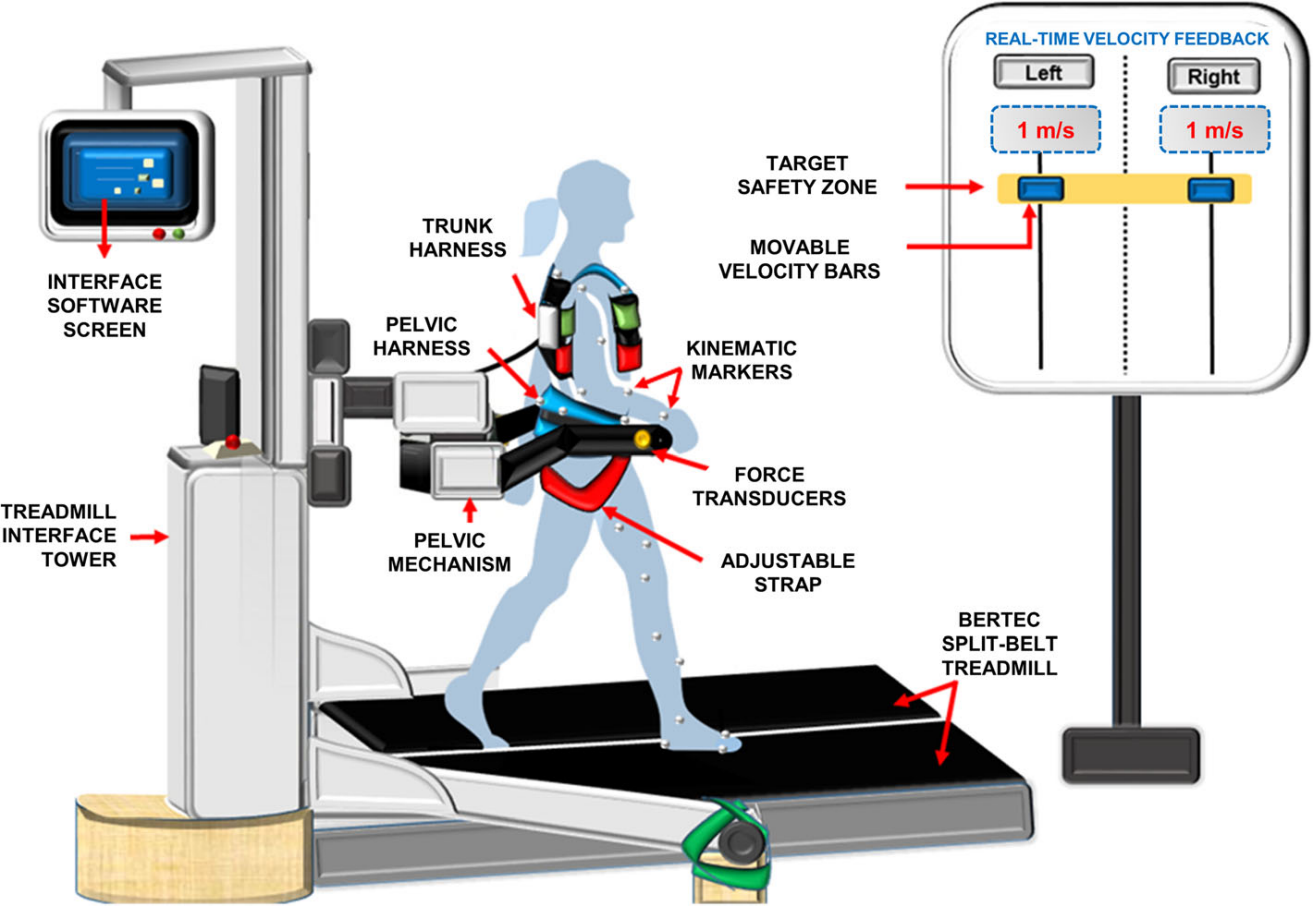
Participant walking in the KA treadmill interface using visual feedback via the interface’s software output to target 1.0 +/− 0.2 m/s highlighted by a yellow target zone and projected on a screen (at eye level), with placement of kinematic markers shown for one limb

We used the environment of an intent-driven robotic treadmill interface that consists of the KineAssist™ (KA) robotic device [30] (HDT Global, Solon OH) synced to a dual-belt, force plate-instrumented Bertec treadmill (Fig. 1) (BERTEC, Columbus, OH, USA).

We have previously published detailed descriptions of the control mechanics, walking biomechanics, and energetics in this device in both healthy-nonimpaired individuals and individuals poststroke [31–35]. Briefly, this interface consists of a pelvic mechanism with a pelvic harness that secures participants walking inside it through adjustable cloth straps around the waist and hips. The pelvic mechanism allows minimally impeded movement in the vertical, horizontal, and medio-lateral planes, providing a total of six degrees of freedom. However, for this study, we locked the pelvic mechanism to allow COM movement only in the fore-aft (relative to treadmill belts) and vertical directions of interest, to limit the effects of off-axis forces on propulsion generation.

A separate adjustable trunk harness, connected to the pelvic mechanism, secured the participant’s trunk allowing forward-backward trunk tilting, but preventing excessive forward lean. Two bidirectional force transducers located at the height of each hip (in the pelvic harness) sensed the net forces generated by the body and applied through the hip/pelvis interface, to control belt speed (as described in the next section). These optical encoders also tracked the vertical height of the mechanism and triggered a “safety-catch” feature, by sensing any drop in the pelvic mechanism height to “catch” the participant walking inside the device (at a preset height) thereby preventing a fall to the treadmill surface. Since the robotic system locked participants at a specific location on the treadmill belt, it prevented them from traveling forward or backward off the treadmill belts. These collective features rendered our experimental environment safe for evaluation of self-controlled walking speed function in different gait conditions.

### Force-velocity relationship of KA split-belt treadmill interface

Participants walking inside the interface could control their walking speed using the interface’s force-velocity relationship, which allowed the investigator to set the required minimum net fore-aft force magnitude that participants had to generate at the hip to initiate the treadmill belt motion. Once the belt started moving, participants had to increase their fore-aft force magnitude in order to attain and maintain a steady-state walking speed. The two hip force transducers recorded and relayed these net forces to the main control system using a closed-loop haptic control algorithm, enabling participants to dictate the speed of each belt on a step-by-step basis, making the system “intent-driven” or “self-driven”. We have previously shown that walking biomechanics in this environment are similar to typical treadmill walking [31, 34].

### Application of different fore-aft resistance levels at a target speed of 1.0 m/s

For this experiment, we selected a target walking speed of 1.0 m/s +/− 0.2 m/s i.e., target speed range, to account for the sinusoidal nature of normal walking. We selected 1.0 m/s as it is close to the comfortable overground speed of healthy-nonimpaired adults, and our previous research involving walking in the KineAssist has demonstrated that a slightly slower than comfortable speed is desirable for quick adjustments to this walking environment [18, 34, 36, 37]. However, in the future we could scale the FA resistance levels to any selected speed based on the system’s force-velocity relationship (see Eq. 1 below). Similar to the Gottschall and Kram study [18], we chose four FA resistance levels (10, 15, 20, and 25%) taken as percentages of a participant’s vertical body weight. We used an algorithm that accounted for the participant’s vertical body weight, interface control parameters, and the system’s force-velocity relationship to calculate their target resistance levels. Thus, we normalized FA resistance by body weight for the same target speed using.

1 $$ \boldsymbol{b}=\boldsymbol{y}\hbox{-} \boldsymbol{mx} $$

Where **b** is the fore-aft resistance (Newtons) to move the belt at an intended speed, **y** is the percentage of vertical body weight (Newtons) needed to maintain treadmill belt movement at 1.0 m/s, **m** is the sensitivity constant of the interface (set for all participants at 50 N-sec/m that allowed quick response of the belt with minimum delay (0.01 m/s)), and **x** is the target self-driven/self-controlled velocity of the tied-treadmill belts (1.0 m/s) (Example provided in the supplemental section demonstrates FA resistance level calculations for one participant using the force-velocity relationship). In summary, we were able to calculate the proper magnitudes of FA resistance for each individual participant, regardless of body weight, that they had to overcome to maintain a target speed of 1.0 m/s.

### Visual feedback

To ensure that participants maintained their target speed of 1.0 m/s, we provided real-time visual feedback of the tied treadmill-belt speed using the KA interface software. We projected the visual feedback onto a 5 × 6 foot projector screen, placed five feet from the treadmill interface (Fig. 1). We highlighted the target-speed zone in a yellow block (displayed at eye level) and instructed participants to maintain their speed in the “yellow” zone i.e., 1.0 m/s ± 0.2 (SD), as walking is a cyclical motion and will fluctuate sinusoidally around a mean. We did not specifically screen participants for visual impairments prior to study participation; however, we verbally confirmed that all participants were able to easily see the target and carefully evaluated speed target maintenance in our results to ensure that participants were indeed capable of maintaining this target.

### Data trials

After a suitable warm-up and familiarization period, participants completed four randomized FA resistance trials at a target speed of 1.0 m/s inside our interface. We collected a minimum of 30 consecutive strides for each resistance level only after visually confirming that participants were able to achieve a steady-state gait pattern, by maintaining their target speed consistently for ten seconds. As we were mainly interested in short-term limb changes to FA resistance, each experimental trial was 40 to 60 s long, to enable participants to maintain target speed (initial few strides) and collect 30 strides (minimum) per limb as other biomechanical studies have also done [37–39]. We continuously monitored heart rate using a GARMIN wrist monitor and provided participants with minimally 30-s rest breaks after each resistance trial. We monitored heart rate carefully for safety purposes only and ensured that it did not exceed 80% heart rate reserve (HRR; based on Karvonen formula [40]) during all trials. We also used heart rate recovery to ensure that participants were recovered enough to commence the next trial (heart rate value below 50% HRR).

### Overview of measures

**Fig. 2:**
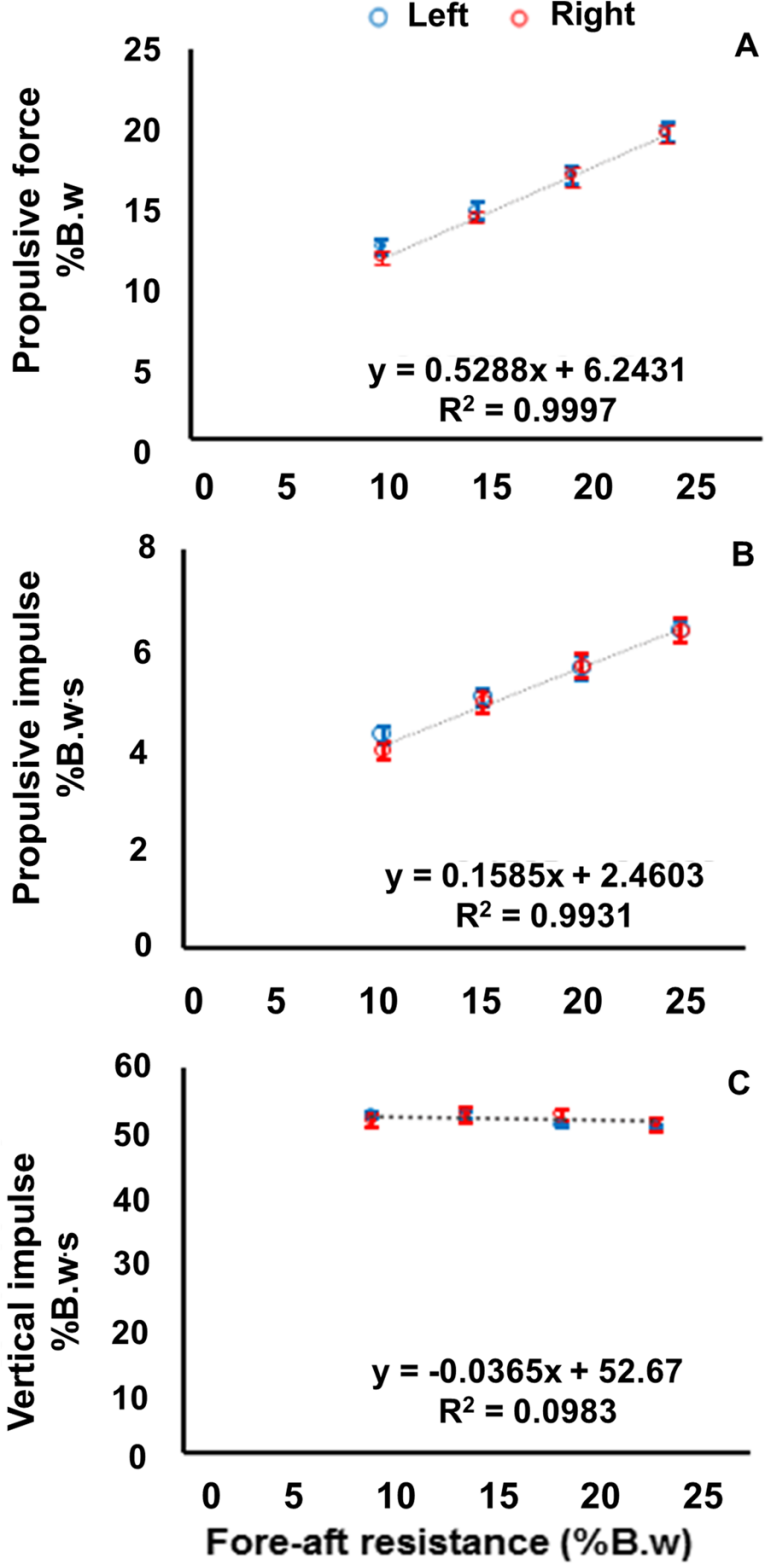
Mean propulsive force (% B.w.) (**a**); and mean propulsive impulse (% B.w∙s) (**b**) both during 50 to 100% of the stance phase; and mean vertical impulse (% B.w∙s) (**c**) during 0 to 100% of the stance phase across all fore-aft resistance trials. Note: error bars are standard error

We primarily assessed propulsive impulse i.e., the time integral of the positive fore-aft GRF during the second half of stance; (Pimpulse (PI_50_) = ∫Fore-aft GRF × dt (50% stance)) i.e., period of propulsion corresponding to ankle “push-off” from 50% of stance to toe off (Fig. 2b). We also measured mean propulsive force (second half of stance) to ensure that changes in stance time were not responsible for increases in propulsive impulse, along with stance time and stride time across trials. Additionally, we assessed vertical impulse, (Vimpulse = ∫vertical GRF × dt), during the entire stance phase (i.e., heel strike to toe off) to ensure that FA resistance was only affecting fore-aft loading and not modifying vertical loading or force generation. As propulsion is associated with limb angle changes, we measured leading and trailing limb angle during stance. As supplementary measures, we calculated positive joint work for each joint and total positive work for the dominant limb, during the entire gait cycle, per FA resistance trial.

### Data acquisition

We collected individual-limb GRFs via the Bertec instrumented treadmill, with kinetic data sampled at 1000 Hz. We also collected 3D kinematic data, sampled at 100 Hz, using an eight-camera Qualisys motion capture system (Qualisys Inc., Gothenburg, Sweden). We used a custom 36 passive-reflective markers setup with each marker being 1-cm in diameter. Markers were placed bilaterally as follows: acromion processes, manubrium of sternum, thoracic spine level with sternum, ASIS, PSIS, sacrum, midline and lateral thighs, midline and lateral shanks, lateral malleoli, first and fifth digits, and calcaneus (see example marker set-up in Fig. 1) [35]. We collected the real-time velocity of each treadmill belt and forces applied to the pelvic-mechanism force transducers (100 Hz) using our treadmill-interface’s custom software.

### Biomechanical data processing

We processed all data using custom MATLAB scripts (Mathworks®, version R2016b), and calculated all kinetic and kinematic variables either over the stance phase (ipsilateral heel strike to ipsilateral toe off) or over a complete gait cycle (ipsilateral heel strike to ipsilateral heel strike). We filtered all data using a low-pass Butterworth filter at a cutoff frequency of 15 Hz (kinetic) and 8 Hz (kinematic), respectively. We used Visual 3D (C-Motion, Germantown, MD, USA) to obtain joint powers for work calculations, but performed all kinematic data post processing via MATLAB using kinetic gait events (heel strikes and toe-offs with a threshold of 1.5% body weight per limb) [34, 41]. We included an average of 30 consecutive strides per limb per participant for each FA resistance condition.

### Kinetic gait variables

We normalized all kinetic (GRF) data to each participant’s body weight. We calculated all joint powers as the net muscle moment and joint angular velocity product (P = M × ω). For mechanical work, we normalized and integrated all joint powers (to body mass (W/kg)) during the entire gait cycle using the formula, W = ∫ P × dt (J/kg). All positive work values indicate power generation and negative work values indicate power absorption.

### Statistical analyses

We used SPSS (22 version) for all statistical analyses and checked that all primary and secondary dependent measures were normally distributed (Shapiro-Wilk’s > 0.05). For all kinetic GRF variables, to assess increasing or decreasing treads in propulsive- and vertical-force generation, we compared individual participant’s (dominant limb) linear slope relationships across all FA resistance levels to zero using one-sample *t*-tests. For example, if an individual had no increase in propulsion across FA resistance levels, they would have a slope coefficient of zero. A positive slope that is significantly different from 0 indicates an increasing relationship between FA resistance and propulsion output, and similarly a negative slope significantly different from 0 would indicate a decreasing relationship with increasing FA resistance. We used separate one-way repeated measure’s ANOVAs (repeated across resistance levels) for all secondary spatiotemporal variables, i.e., stance time, stride time, limb angles, and individual belt speed (control variable), along with individual positive ankle, knee, and hip joint work and total positive lower-limb work across joints. We used *p ≤* 0.05 to determine significance, with Greenhouse-Geisser corrections for violations of sphericity and Bonferroni corrections for multiple post-hoc comparisons. We did not observe any effects of gender or height on these dependent variables and therefore did not include them in our final statistical models. For visual aid and interpretation, we provide ensemble average profiles for vertical and fore-aft GRFs during stance and joint powers during the whole gait cycle.

## Results

### Control over walking speed across FA resistance levels

We observed significant differences in tied-belt speed across FA resistance levels [F (3.45) =5.96, η^2^ *=* 0.28*, p* < 0.05)]. However, observed speeds were still within the a priori acceptable target speed zone (1.0 ± 0.2 m/s) (Table 1). Thus, we did not consider these results practically significant as values were within the expected standard deviation range provided to participants during all walking trials via visual feedback.

**Table 1:** Spatiotemporal variables for the dominant limb (Mean + 95% CI) across all four fore-aft (FA) resistance levels

FA resistance level	10%	15%	20%	25%
Tied belt velocity (m/s)	1.13 [1.11 to 1.16]	1.06^(1)^ [1.02 to 1.11]	1.06^(1)^ [1.03 to 1.08]	1.06^(1)^ [1.02 to 1.09]
Stance time (s)	0.69 [0.66 to 0.72]	0.69 [0.66 to 0.72]	0.67 [0.64 to 0.70]	0.65 ^(2)^ [0.63 to 0.68]
Stride time (s)	1.05 [1.00 to 1.09]	1.05 [1.00 to 1.11]	1.02 [0.96 to 1.08]	1.00 [0.95 to 1.06]
Trailing limb angle (^o^)	24.3 [22.90 to 25.69]	25.4 [23.98 to 26.72]	26.7^(1)^ [24.86 to 28.52]	27.4^(1)^ [24.98 to 29.75]
Leading limb angle (^o^)	−18.4 [−20.7 to −15.97]	−17.6 [−19.6 to − 15.48]	−15.6^(2)^ [− 17.8 to − 3.31]	−12.6^(4)^ [− 16.6 to − 8.49]

Note: All superscripts represent significant post-hoc pairwise comparisons with Bonferroni corrections for each measure

### Primary results regarding propulsion across fore-aft force levels

All participants increased their propulsive-force generation across FA resistance levels, evidenced by significant increases in the slopes compared to zero: mean propulsive forces (mean slope = 2.45, SD = 0.7, *t* (16) =14.44, *p* < 0.01) (Fig. 2a); and mean propulsive impulses (mean slope = 0.71, SD = 0.25, *t* (16) = 11.81, *p* < 0.01) (Fig. 2b)**.** However, the slopes across mean vertical impulses (mean slope = − 0.04, SD =0.17, *p* > 0.05) (Fig. 2c) did not significantly change across FA resistance levels (i.e., were not significantly different from zero).

### Descriptive comparisons of fore-aft and vertical GRF profiles during stance

**Fig. 3:**
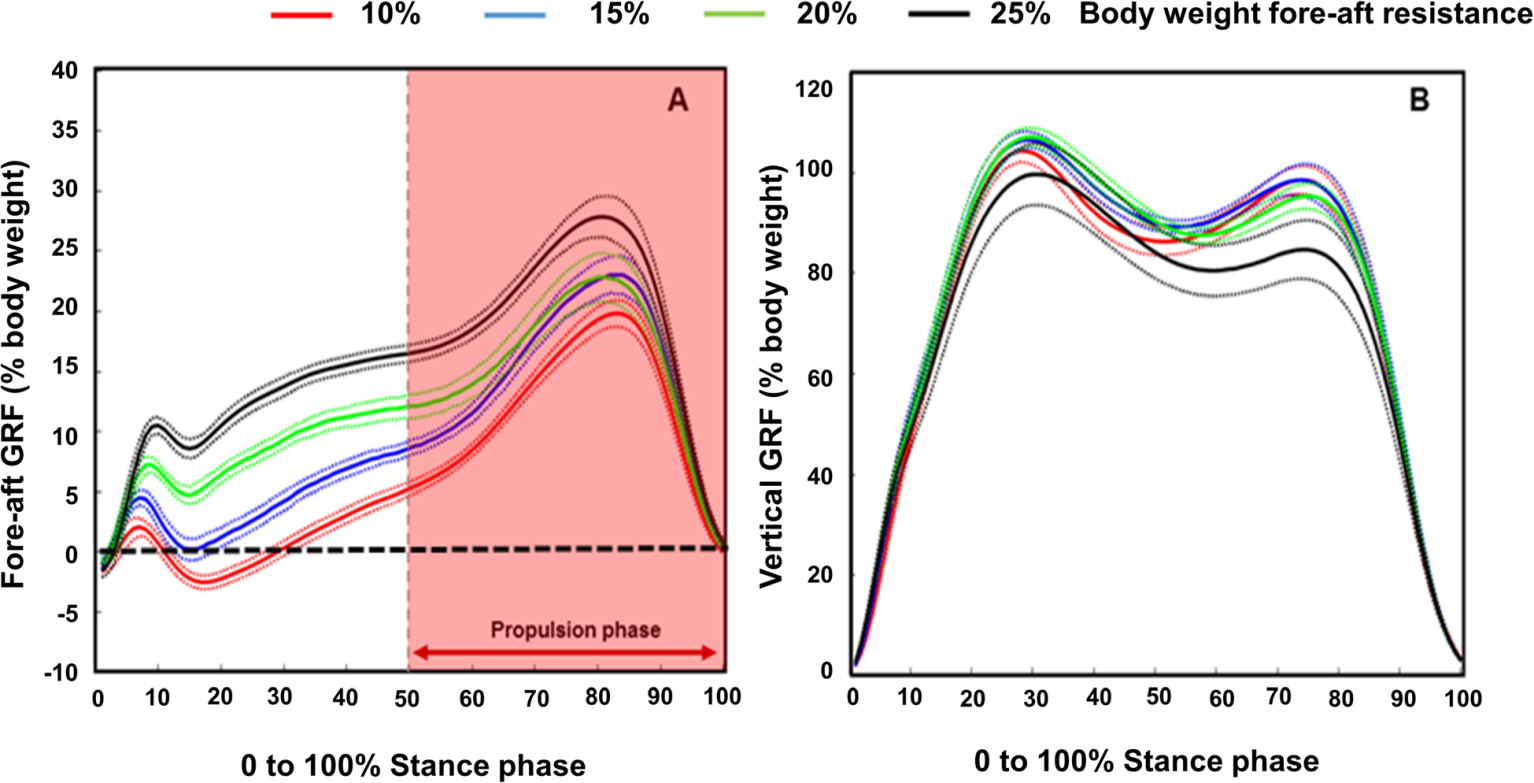
Dominant limb ensemble average (with standard error) fore-aft GRF profiles (**a**); and ensemble average (with standard error) vertical GRF profiles (**b**) across all FA resistance conditions at 1.0 m/s target velocity. Propulsion phase (shaded area in A) highlights the second half of the stance phase used for propulsion calculations. Note red = 10%, blue =15%, green = 20% and black =25% B.w. fore-aft resistance levels

The average ensemble vertical GRF profiles remained relatively the same for 10, 15, and 20% FA resistance trials. During the 25% trial, the first vertical peak decreased slightly with a more visible decrease in the second vertical GRF (Fig. 3b). On comparison of the fore-aft profiles (Fig. 3a), we found that participants decreased braking-force production and had a larger propulsive phase at 10% FA resistance.

At subsequent FA resistance levels (15 to 25%), participants exhibited no braking-force generation, and propelled during the entire stance phase, increasing their fore-aft ensemble average magnitudes. To account for the variation in timing of propulsion across FA resistance levels, we focused propulsive-force calculations to the second half of stance i.e., the propulsion phase of walking.

### Spatiotemporal variables

We did not observe any significant changes in mean stance time or mean stride time across FA resistance levels (*p* > 0.05). However, trailing limb angles increased with increasing FA resistance, evidenced by a main effect of resistance (F (1.97, 27.7) = 7.6, *p <* 0.05 η^2^ = 0.4). Post hoc comparisons revealed significant increases at 20 and 25% FA resistance levels compared to 10%, with no significant post-hoc differences between any other FA resistance levels (*p* > 0.05). Additionally, leading limb angles decreased significantly with increasing FA resistance, evidenced by a main effect of resistance (F (1.37,19.25) = 5.85, *p <* 0.05, η^2^ = 0.6). Post hoc comparisons revealed significant decreases from 10 to 20% and from 10 to 25% FA resistance, with no significant post-hoc differences between other FA resistance levels (*p* > 0.05).

### Description of joint powers across FA resistance levels

**Fig. 4:**
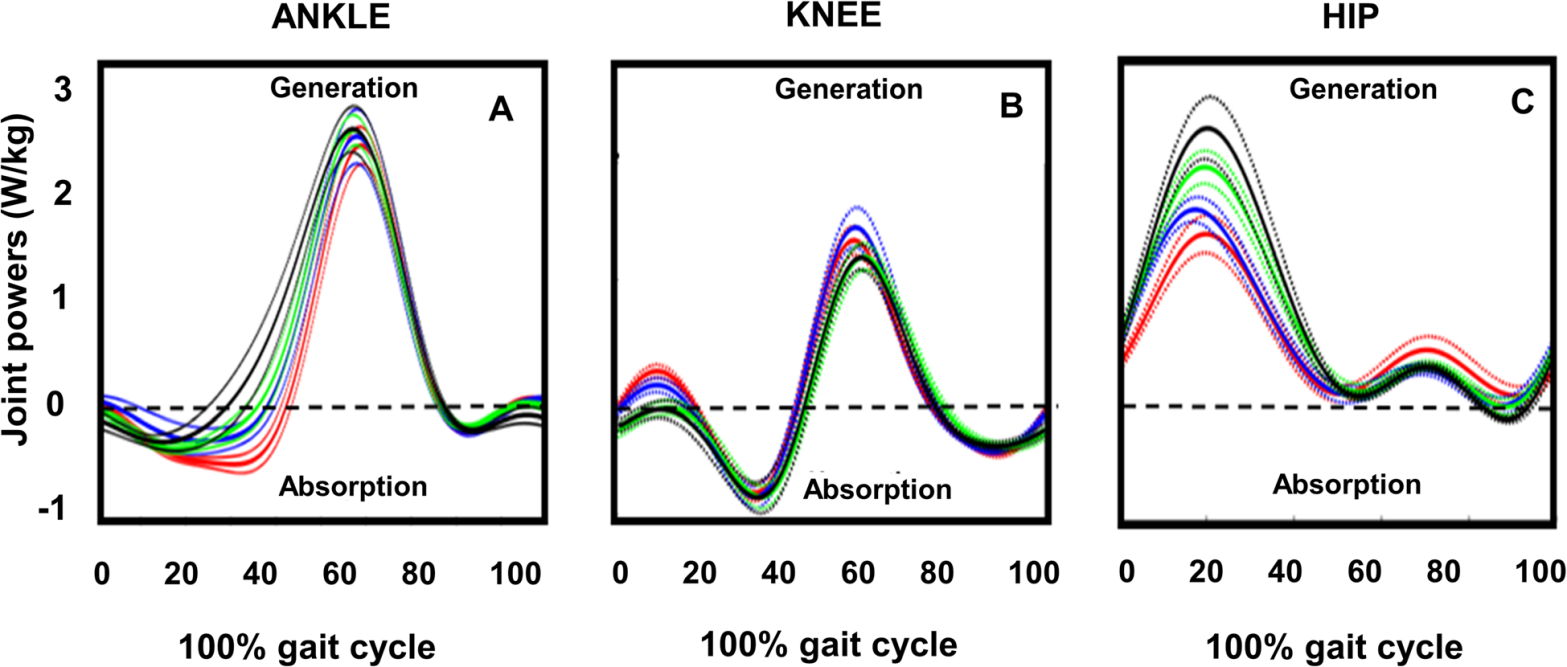
Ensemble average profiles of joint powers with standard error (dotted colored lines) for ankle (**a**), knee (**b**), and hip (**c**), respectively, at 10% (red), 15% (blue), 20% (green), and 25% (black) fore-aft (FA) resistance across 0 to 100% of the full gait cycle

Magnitude of ankle power (Fig. 4a) absorption decreased at initial stance with increases in peak ankle power and ankle power generation during the propulsion phase. Knee power generation (Fig. 4b) was fairly consistent across FA resistance levels. Regarding the hip joint, power generation magnitude markedly increased with the majority of power generation occurring during initial stance (Fig. 4c).

### Work done across lower-limb joints against FA resistance

**Fig. 5:**
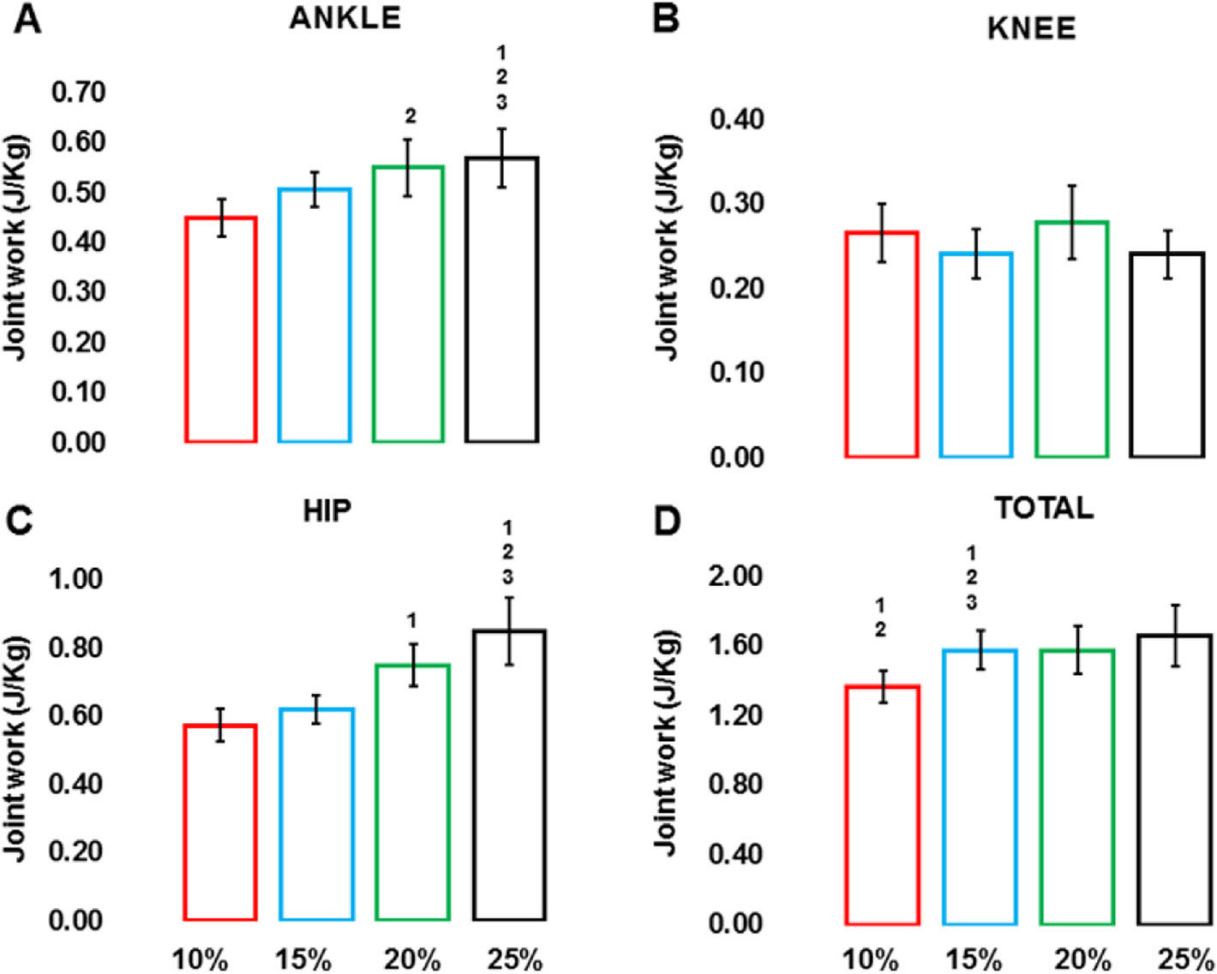
Mean and standard error values for positive work at the ankle (**a**), knee (**b**), and hip joint (**c**), and total positive work across all three joints (**d**) for all four fore-aft (FA) resistance levels (10% = red, 15% = blue, 20% = green, 25% = black conditions). Note: superscripts represent significant pairwise comparisons with Bonferroni corrections at *p* < 0.01, 1 = 10%, 2 = 15%, 3 = 20%, and 4 = 25% FA resistance levels

Positive work increased at the ankle and hip joints and the sum across the joints with increasing resistance level (Fig. 5). Positive work at the ankle increased with increasing FA resistance, evidenced by a main effect of resistance (F (3, 45) = 11.34, *p* ≤ 0.001, η^2^ = 0.7) (Fig. 5a). We only found significant post-hoc differences at 15% (0.5 J/kg 95% CI [0.42–0.57]), 20% (0.58 J/kg 95% CI [0.49–0.66]), and 25% (0.6 J/kg 95% CI [0.5–0.69]) levels compared to 10% FA resistance (0.4 J/kg 95% CI [0.36–0.51]), while all other post-hoc comparisons between FA resistance levels were not significantly different (*p* > 0.05).

We did not find any significant increase in positive work at the knee across FA resistance conditions (*p* > 0.05) (Fig. 5b). Positive work at the hip increased with increasing FA resistance (Fig. 5c), evidenced by a main effect of FA resistance (F (3, 45) = 29.29, *p* ≤ 0.001, η^2^ = 0.66), with significant post-hoc differences between 10% (0.59 J/kg 95% CI [0.5–0.68]), 15% (0.64 J/kg 95% CI [0.56–0.72]), 20% (0.79 J/kg 95% CI [0.65–0.93]), and 25% (0.94 J/kg 95% CI [0.79–1.09]) FA resistance levels. All other post-hoc comparisons were not significant (*p* > 0.05).

Total positive work across all three joints also increased with increasing FA resistance (Fig. 5d), evidenced by a main effect of resistance (F (1.7, 26) = 16.88, *p* ≤ 0.001, η^2^ = 0.5). We found significant post-hoc differences between 10% (1.3 J/K 95% CI [1.19–1.5]) and 20% (1.6 J/kg 95% CI [1.36–1.8]), and 10 and 25% FA resistance (1.77 J/kg 95% CI [1.53–2]) respectively. However, we did not find any significant post-hoc effects at 15% (1.6 J/kg 95% CI [1.37–1.83]) compared to 10% FA resistance.

## Discussion

Taking advantage of a unique robotic treadmill interface, we explored the effects of increasing fore-aft loading during walking at a self-controlled target speed of 1 m/s. We hypothesized that participants would increase their interlimb propulsion to maintain a target speed against increasing FA resistance. In support of our hypothesis, healthy-nonimpaired participants proportionately increased intralimb propulsion, without altering vertical limb loading, in order to maintain walking speed in response to greater amounts of applied FA resistance. In addition, we also supported our hypothesis that walking against greater FA resistance would result in increased trailing limb angles and positive joint work. Studies examining resistance and uphill walking at constant speeds have found healthy-nonimpaired individuals tend to scale their peak propulsion forces and duration of propulsion based on the amount of resistance or level of inclination against which they are walking [19, 21, 24, 26, 42, 43]. However, walking on an incline requires greater lower-limb joint range of motion that may not be achievable for an individual with neurological impairments that cause unilateral limb-weakness such as hemiparesis. Walking on a level surface against increasing resistance mitigates the range of motion requirements of hill walking but places similar demands on muscular output.

Our results suggest that the requirements for maintaining a constant target speed against environmental factors that impede forward progression (e.g., FA resistance) possibly facilitates increases in proprioceptive limb-extensor feedback along with feedforward mechanisms to increase propulsion generation. We also observed little to no braking-force generation during initial stance, especially with greater levels of resistance [41, 44]. We acknowledge that walking within our robotic interface provides some attenuation of braking-force generation [31, 34] due to the pelvic-mechanism holding participants in place and limiting forward-backward translation of the COM that occurs during typical walking. This effect coupled with requirements of walking against FA resistance may have further reduced braking. Walking against FA resistance is similar to uphill walking [25, 42], which has also shown reductions in braking-force generation, possibly due to increasing demands of raising the COM and earlier need for propulsion generation to maintain target speeds.

Regarding limb angle changes, participants increased their trailing limb angles and decreased their leading limb angles at higher FA resistance levels. Several studies have indicated that an increase in trailing limb angle is a strategy to increase propulsive-force generation [28, 45–48], while reduction in leading limb angle is also indicative of participants trying to quickly get the limb into a more posterior position to propel the COM forward. We believe that such a strategy enabled participants to increase rate and magnitude of propulsive-force generation to meet the demands of greater resistance and maintain walking speed, as stance time and stride time did not significantly change across conditions. These findings are consistent with studies that highlight how increases in trailing limb angle are associated with increases in propulsion needed to attain faster walking speeds [26, 38, 41, 49].

At the individual joint level, walking against greater FA resistance resulted in increased ankle and hip power generation with little to no changes in knee power generation. Collectively, these changes indicated use of an ankle and hip strategy to attain target walking speeds against higher FA resistance levels [24, 42, 50]. Visual analysis of joint moments (not reported here) also revealed increases in positive hip joint moments at higher resistance levels, with minimal to little change in ankle and knee joint moments. This suggests that an increase in ankle joint angular velocity facilitated the increased ankle power generation, while an increase in hip joint force production (moment) facilitated the increased hip power production. This strategy implies that perhaps the larger hip muscles were best suited to lend themselves to the increased demands of fore-aft limb loading to maintain target speed inside the treadmill interface. It has been reported that positive hip joint powers are known to increase significantly over the ankle at faster walking speeds [51, 52]. We found similar joint changes in our study, possibly to move the limb in a position directly underneath and behind the body to increase forward propulsion of the COM. Additionally, we visually noted an absence of hip joint power absorption that typically occurs in terminal stance and is associated with stretching of hip proprioceptors to facilitate offloading to initiate swing. This lack of negative hip work during terminal stance might indicate that walking against FA resistance created a different type of proprioceptive feedback (i.e., load-related instead of stretch-related) to modulate limb offloading to enable propulsive-force generation to maintain target speed Additional file 1.

### Limitations

In this study, we only explored walking function against FA resistance at one constant speed (1.0 m/s). However, prior published research from our lab has explored effects of FA resistance at different speeds, albeit in different experimental conditions, in both healthy-nonimpaired and poststroke populations [11, 31, 34, 35]. We also did not assess a true “no-load” condition (i.e., 0% FA resistance) due to the minimum resistance requirements of the self-driven treadmill’s force-velocity relationship. However, we were primarily interested in the changes in propulsion across increasing FA resistance levels, not necessarily between 0% resistance versus a particular level of resistance. It is possible that the nature of the visual feedback provided for speed maintenance in our study may have had effects not accounted for here on propulsion-force generation. However, we chose to control speed across participants in order to observe changes in propulsive-force output due to increasing FA resistance levels; thus, visual feedback was necessary. We did not report hip-transducer load values, as they are simply the loads applied by the participant against the pelvic-harness to overcome the applied fore-aft resistance. These values differ across all participants (resistance was scaled based on body weight), and would show a linear increase in relation to the fore-aft resistance applied, accounted by the force-velocity relationship driving our treadmill interface. Thus, these values would not confirm whether participants were selectively using a hip strategy while walking. We cannot discount the role of our device in influencing the strategy participants chose to employ while walking against resistance. However, we do not feel that the hip strategy observed is solely due to our device given results from similar studies and paradigms (e.g., hill walking) that also showed larger positive work at the hip and ankle compared to the knee [21, 24, 26, 42, 43]. Lastly, while kinetic and kinematic variables help in determining muscle-force generation strategies, they only provide pure mechanical measurements. Future studies should also measure EMG responses of plantarflexors (e.g., gastrocnemius, soleus), which are primarily associated with propulsion along with kinetic and kinematic changes to gain more complete insight into proprioceptive changes and the neuromechanical impact of FA resistance [9].

## Conclusions

We demonstrated that walking against FA resistance, applied by a robotic system that allowed people to control their own speed, proportionately increased fore-aft limb loading without significant changes in vertical limb loading. The experimental environment of the robotic treadmill interface enabled us to manipulate the fore-aft loading demands during stance while participants controlled their walking speeds. Our results suggested that FA resistance can be applied in environments that allow self-controlled walking in order to increase propulsive force output of healthy-nonimpaired individuals.

## Future work

Future studies will evaluate whether this approach may be a useful rehabilitation application, especially for individuals who may have difficulty walking on inclines or at fast speeds. For example, this approach could be used for ambulatory individuals with hemiparesis both as a measurement tool to assess functional strength related to walking (i.e., maximum propulsion capability or propulsion “reserve”), and also serve as a training environment for individuals who are able to increase propulsion under increased FA loading. Individuals poststroke may generate forward propulsion by employing a hip extension torque strategy during stance, given that ankle plantarflexor strength is frequently impaired poststroke [48]. The nonimpaired participants in this study largely relied on a hip strategy to maintain walking speed against added FA resistance; thus, individuals poststroke may employ a similar strategy in this walking environment. FA resistance offers a mechanism to increase workload at any constant speed (e.g., an individual’s self-selected comfortable speed), as opposed to using progressively faster walking speeds that individuals with hemiparesis may not be able to attain [53–56]. We used 1.0 m/s in the present study since this speed is close to the comfortable speed for healthy-nonimpaired adults, but as our FA resistance equation demonstrates, resistance can be scaled to be appropriate for a range of walking speeds. Additionally, level walking under FA resistance may be a more realistic option than walking on an incline for individuals with hemiparesis, since inclined walking requires greater lower-limb joint range of motion [24, 42, 50, 57] that may not be achievable for these individuals. Walking on a level surface against increasing resistance mitigates the range of motion requirements of hill walking but achieves similar demands on muscular output.

## Additional file

Additional file 1: Fore-velocity relationship calculation for fore-aft resistance (Newton) for one participant based on body weight (Newton) and target speed (m/s). (DOCX 20 kb)

## Data Availability

Available.
