# Supplementary material for: Fore-aft resistance applied at the center of mass using a novel robotic interface proportionately increases propulsive force generation in healthy nonimpaired individuals walking at a constant speed
Source: J Neuroeng Rehabil. 2019 Sep 6;16:111. doi: 10.1186/s12984-019-0577-x (PMC6731616; doi:10.1186/s12984-019-0577-x)
Supplement: Supplementary file 1 — Fore-velocity relationship calculation for fore-aft resistance (Newton) for one participant based on body weight (Newton) and target speed (m/s). (DOCX 20 kb) [file 12984_2019_577_MOESM1_ESM.docx]

SUPPLEMENTAL DATA

**Example of KA-interface force-velocity relationship**: An individual weighing 135 lbs is 600 Newtons (135*4.448 N). Therefore, fore-aft resistance equivalent to 10% vertical body weight while walking at a target speed of 1m/s will

b= (10/100)*600–(50N.m/s)(1m/s) = 10 N (Supplemental)


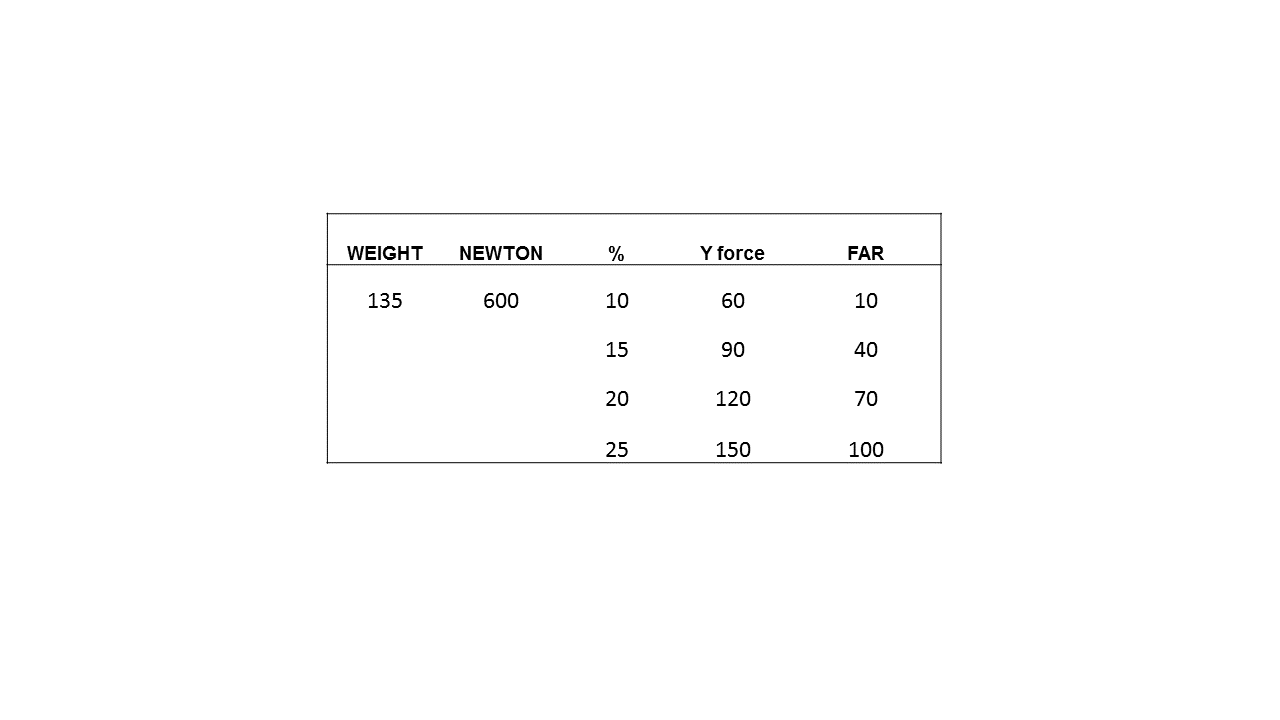


*FAR = Fore-aft resistance
